# The Impact of Autonomic Nervous System Modulation on Heart Rate Variability and Musculoskeletal Manifestations in Chronic Neck Pain: A Double-Blind Randomized Clinical Trial

**DOI:** 10.3390/jcm14010153

**Published:** 2024-12-30

**Authors:** Hani A. Alkhawajah, Ali M. Y. Alshami, Ali M. Albarrati

**Affiliations:** 1 Department of Physiotherapy, King Fahd Hospital of the University, Imam Abdulrahman Bin Faisal University, P.O. Box 40244, Khobar 31952, Saudi Arabia; 2 Department of Physical Therapy, College of Applied Medical Sciences, Imam Abdulrahman Bin Faisal University, P.O. Box 2435, Dammam 31441, Saudi Arabia; alshami@iau.edu.sa; 3 Rehabilitation Health Sciences, College of Applied Medical Sciences, King Saud University, P.O. Box 10219, Riyadh 11451, Saudi Arabia; albarrati@ksu.edu.sa; 4 King Salman Center for Disability Research, Riyadh 1164, Saudi Arabia

**Keywords:** vagus nerve stimulation, heart rate variability biofeedback, sympathetic nervous system, parasympathetic nervous system

## Abstract

**Background:** The role of autonomic nervous system (ANS) modulation in chronic neck pain remains elusive. Transcutaneous vagus nerve stimulation (t-VNS) provides a novel, non-invasive means of potentially mitigating chronic neck pain. This study aimed to assess the effects of ANS modulation on heart rate variability (HRV), pain perception, and neck disability. **Methods:** In this double-blind randomized clinical trial, 102 participants with chronic neck pain were randomly allocated to one of three groups: t-VNS plus standard-care physiotherapy (SC-PT), heart rate variability biofeedback (HRV-BF) with SC-PT, or SC-PT alone. Interventions were administered three times weekly for 6 weeks. The following outcome measures were assessed at baseline and after 6 weeks: HRV, the visual analog scale (VAS), the pressure pain threshold (PPT), and the neck disability index (NDI). **Results:** The t-VNS group exhibited significant improvements compared to the HRV-BF and SC-PT groups. Specifically, t-VNS increased the RR interval (mean difference [MD] = 35.0 ms; *p* = 0.037) and decreased the average heart rate (MD = −5.4 bpm; *p* = 0.039). Additionally, t-VNS reduced the VAS scores (versus HRV-BF: MD = −0.8 cm, *p* = 0.044; SC-PT: MD = −0.9 cm, *p* = 0.018), increased the PPT (versus HRV-BF: MD = 94.4 kPa, *p* < 0.001; SC-PT (MD = 56.2 kPa, *p* = 0.001)), and lowered the NDI scores (versus HRV-BF: MD = −4.0, *p* = 0.015; SC-PT: MD = −5.9, *p* < 0.001). **Conclusions:** t-VNS demonstrated superior effectiveness compared to HRV-BF and SC-PT in regulating HRV, alleviating pain, and enhancing functional capabilities in individuals with chronic neck pain.

## 1. Introduction

Neck pain is a common musculoskeletal condition that affects the cervical spine and may radiate to one or both upper limbs [1]. It is classified as nonspecific when no identifiable underlying disease or anatomical abnormality is present [2]. In 2017, the Global Burden of Disease Study revealed that the age-standardized point prevalence of neck pain was 3551 per 100,000 people, with an annual incidence of 807/100,000 people [1]. Approximately 60% of the individuals experience recurrent episodes of neck pain [3]. Data on the prevalence of neck pain in Saudi Arabia are limited. However, Alshami [4] conducted a retrospective study at a university hospital to establish the prevalence of spinal disorders over 3 years and found that at least 61% of patients referred for neck disorders experienced neck pain.

Clinical practice guidelines recommend several interventions for chronic neck pain, including multimodal approaches to thoracic and/or cervical mobilization/manipulation [5], mixed cervical stretching and strengthening exercises, dry needling, lasers, and intermittent traction [6]. Recent clinical guidelines recommend a combination of exercise and manual therapy as the preferred evidence-based treatment for patients with neck pain, with other treatment options lacking clear evidence [7]. Respiratory exercises appear to be effective for patients with neck pain. A pilot study by Mohan et al. [8] compared the effectiveness of respiratory exercises versus routine physiotherapy exercises and found that patients who received respiratory exercises experienced less pain intensity. The diversity of treatment options for patients with musculoskeletal disorders, including neck pain, may be attributed to different pain mechanisms, namely nociceptive, neuropathic, nociplastic, or autonomic [9,10]. While neck pain is commonly attributed to somatic issues, visceral dysfunction can contribute to nonspecific neck pain, often referred to as visceral referred pain [11]. This occurs when pain originating from internal organs, such as the heart, lungs, or gastrointestinal tract, is perceived in the neck due to shared neural pathways between these organs and neck tissues [11]. The autonomic nervous system (ANS) plays a significant role in both the initiation and persistence of musculoskeletal pain. The sympathetic nervous system, a branch of the ANS, is activated in response to pain, leading to increased muscle tension, inflammation, and reduced blood flow to the area; these factors contribute to pain [12]. There is evidence that therapies targeting the ANS, such as biofeedback, yoga, and electrical stimulation, can help reduce chronic musculoskeletal pain [13,14]. Heart rate variability biofeedback (HRV-BF) has been shown to help treat symptoms characterized by ANS aberrations [15], including post-traumatic disorder [16], fibromyalgia [17], chronic low back pain [18], and chronic diseases [19]. Similarly, t-VNS has been shown to modulate HRV in healthy adults [20,21,22]. While other forms of biofeedback, such as electrodermal activity biofeedback, target different physiological parameters, t-VNS and HRV-BF specifically focus on the ANS through HRV. These techniques offer the advantages of being safe, non-invasive, accessible, and user-friendly [23].

To our knowledge, studies investigating the effects of ANS modulation using non-medical techniques, such as vagus nerve stimulation (VNS) or HRV-BF, in patients with chronic neck pain are scarce. Only one pilot study has investigated the impact of HRV-BF in patients with stress-related chronic neck and shoulder pain [24]. The authors found that HRV-BF improved perceived health, resting heart rate (HR), and reactivity to hand grip and cold pressors; however, no improvements were noted in neck pain and function or stress-related symptoms [24]. This study aimed to investigate the effects of ANS modulation using VNS and HRV-BF on HRV, pain, and neck disability in patients with nonspecific chronic neck pain. We hypothesized that patients with chronic neck pain who receive VNS or HRV-BF training will show greater improvements in HRV, reduced pain intensity, and improved functional abilities than controls receiving only standard physiotherapy care.

## 2. Methodology

### 2.1. Study Design, Setting, and Ethical Considerations

This was a three-arm double-blind randomized clinical trial. The assessor was blinded to the allocation of the treatment groups, and the treating therapists were blinded to the outcome measurements. This study was conducted between June 2023 and December 2023 at the Department of Physiotherapy, King Fahd Hospital of the University, Saudi Arabia. The study was conducted in accordance with the Declaration of Helsinki and followed the Consolidated Standards of Reporting Trials (CONSORT) reporting guidelines [25]. This study was approved by the Institutional Review Board (IRB) of Imam Abdulrahman Bin Faisal University, Dammam, Saudi Arabia (IRB-PGS-2023-03-096), dated 26 February 2023. Eligible patients were fully informed of the risks and benefits and were required to read and sign a written consent form. To ensure participant confidentiality, a code was used instead of the patient’s name on the data collection form. All data collection forms were securely stored in a locker and were accessible only to the researchers. The study protocol was registered at ClinicalTrials.gov (NCT05922059, June 2023).

### 2.2. Sample Size Calculation

Sample size was determined using G*Power 3.1.9.6 software (Fanz Faul, Universität Kiel, Kiel, Germany). The effect size (ES) for HRV was derived from a previous study [24]. The sample size calculation utilized the following parameters: a repeated-measures ANOVA with a within–between interaction, an ES (f) of 0.15, an alpha level of 0.05, a power of 80%, a correlation among repeated measures of 0.5, three groups, three measurement time points, and a non-sphericity correction (ε) of 1. The estimated required sample size was 93 participants. To account for a 10% attrition rate, the final sample size was set at 102 participants (34 per group).

### 2.3. Participants

Participants with a physician-confirmed diagnosis of nonspecific chronic neck pain were recruited consecutively. Patients were included in the study if they were adults aged ≥18 years old, had neck pain for ≥3 months, and reported peak neck pain of >3 on the visual analog scale (VAS) over the previous 24 h [7]. Patients were excluded if they had undergone neck surgery, received an intra-articular corticosteroid injection within 6 months, had current or past (within 4 weeks) oral corticosteroid use to avoid an increase in blood pressure, neurological diseases, altered temperature or pressure perception, pregnancy, cognitive impairment, arm numbness or tingling, cardiac pacemaker or other implanted electronic devices, cardiac arrhythmia, history of myocardial infarction, local ear disorders, or symptomatic orthostatic hypotension. Patients using non-steroidal anti-inflammatory drugs were instructed to discontinue their use for at least 48 h prior to inclusion.

### 2.4. Randomization and Allocation

The participants were recruited consecutively and randomly allocated to Group A (transcutaneous VNS plus standard-care physiotherapy), Group B (HRV-BF plus standard-care physiotherapy), or Group C (standard-care physiotherapy alone). The randomization sequence was computer-generated by the principal researcher using GraphPad (http://www.graphpad.com/, accessed on 17 May 2023). A total of 102 numbers were generated, and each number and its corresponding group assignment were concealed in a sealed, opaque envelope.

### 2.5. Intervention

The treatment protocol, which involved treatment three times a week for 6 weeks (total of 18 sessions), was implemented by experienced physiotherapists, each with more than 10 years of clinical experience, based on the participant’s assigned group.

Auricular transcutaneous VNS (t-VNS) (NEMOS, Cerbomed, Erlangen, Germany) was applied to the left concha of the external ear (cavity) at a stimulation frequency of 30 Hz for half an hour [26] with the participant seated comfortably in an armrest chair. Monophasic rectangular waveforms were used, and the intensity from the device was adjusted below the participant’s pain threshold. The device parameters were adjusted based on evidence-based guidelines and clinical practice recommendations [26,27].

The HRV-BF device (emWave2, model No. 2-01, HeartMath, Boulder Creek, CA, USA) was used to facilitate breathing training by providing real-time biofeedback on physiological indicators. The participants were guided towards achieving coherence, which was visualized on three levels: low (red), medium (blue), and high (green). The participants were comfortably seated in an armrest chair, connected to the equipment, and given an explanation of the procedure. They received visual HRV feedback from the device during breathing training for 15 min in one set [28,29].

The standard-care physiotherapy (SC-PT) in this study was adopted from the recommendations reported in the clinical practice guidelines of the American Physical Therapy Association for neck pain [6]. It involves cervical joint mobilization, strengthening, and stretching exercises. Cervical spine mobilization was performed on the most painful segment while the participant was in a prone position using grade III oscillations at a rate of two per second for 1 min, applied in two sets. For the neck flexion and extension strengthening exercises, the participants were seated on a chair with arm support. The participants were instructed to perform an isometric exercise for neck flexors, completing four repetitions for four sets. They also engaged in cervical extensor strengthening exercises using a red TheraBand, performing four repetitions for four sets. A stretching exercise for the upper trapezius of the painful side was actively performed with a hold for 30 s for four sets while the participant was seated on an armrest chair. The dosage variables for the strengthening and stretching exercises were adopted from an expert consensus on important chronic nonspecific neck pain motor control and segmental exercises [30].

### 2.6. Outcome Measures

An assessor with >10 years of physiotherapy experience collected demographic data and outcome measurements at baseline and 6 weeks after the intervention. The patients were asked not to discuss their treatment experiences with the assessor.

Heart rate variability (HRV). An HRV monitor (Polar, H10 HR sensor, Kempele, Finland) was used to measure the HRV in the resting supine position for 5 min as recommended by the Task Force of the European Society of Cardiology (1996). A sensor belt was secured to the infra-sternal notch, transmitting HRV data in real-time to the Elite HRV 5.5.1 software (Elite HRV In, Asheville, NC, USA). The software implemented artifact rejection techniques to eliminate interference, noise, and other factors that could compromise the integrity of HRV data [31]. The Polar monitor is valid and perfectly reliable (ICC = 1.00), and it is feasible for measuring HRV [32,33]. The output parameters of the Polar monitor used in this study are listed in Table 1.

Visual analog scale (VAS). Current pain intensity was measured using a 10 cm VAS with the endpoints marked as “no pain” and “worst pain imaginable”. The VAS is a valid and highly reliable (ICC = 0.97) measure of neck pain [34].

Pressure pain threshold (PPT). Pain intensity was quantified using a digital pressure algometer (Somedic AB, Farsta, Sweden) equipped with a 1 cm^2^ surface probe and a 40 kPa pressure rate. Measurements were taken at the site of maximum pain. Participants were asked to indicate when the pressure sensation became painful, and the resultant value was recorded. Three measurements were performed, and the average value was recorded for analysis. Following each measurement, a rest period of 20 s was allocated [35]. This measure demonstrated good reliability in chronic neck pain (ICC: 0.83–0.89) [36].

Neck Disability Index (NDI). The is a self-report questionnaire that evaluates the functional status of individuals with neck pain. It comprises ten sections: pain, personal care, weight gain, reading, headache, concentration, work, driving, sleep, and leisure. Each section is rated on a scale of 0 to 5, where 0 indicates “painless” and 5 denotes “the worst pain imaginable”. The questionnaire is interpreted with the following categories of disability: no disability (0–4), mild (5–14), moderate (15–24), severe (25–34), and complete disability (≥35). The Arabic version of the NDI, which has excellent reliability (ICC = 0.96), was used in this study [37].

### 2.7. Statistical Analysis

Data were analyzed using IBM SPSS for Windows version 26.0 (IBM Corp., Armonk, NY, USA). Descriptive analyses of means, standard deviations, and frequencies were used to present the participants’ characteristics. Differences between the groups at baseline were analyzed using a one-way ANOVA for normally distributed data, while the Kruskal–Wallis test was used for non-normally distributed data. An analysis of covariance was performed to identify between-group differences based on the interaction of three groups across two time stages, while non-normally distributed data were addressed using the Quade test. Baseline characteristics, comorbidities, and medications were considered as covariates. The analysis was performed on an intention-to-treat basis; missing values were handled using forward imputation/last observation carried forward. The significance level was set at *p* < 0.05.

## 3. Results

### 3.1. Participant Characteristics

**Figure 1:**
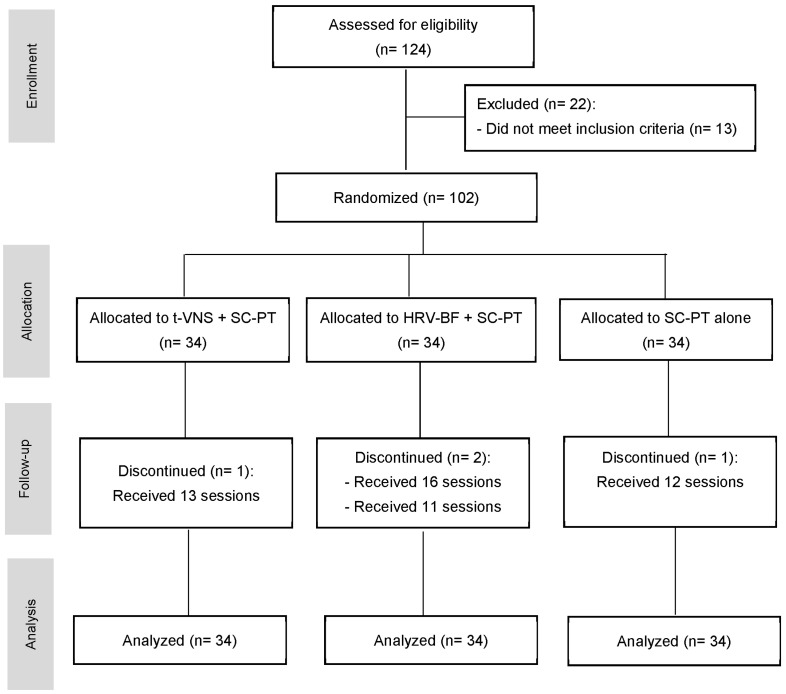
CONSORT diagram of patient enrolment and randomization. t-VNS: transcutaneous vagus nerve stimulation, SC-PT: standard-care physiotherapy, HRV-BF: heart rate variability biofeedback.

In total, 124 newly referred participants were screened for eligibility. Of them, ten participants were excluded because of heart disease, two had recent spine surgery, one scored <3 on the VAS, and nine refused to participate. Thus, a total of 102 participants met the eligibility criteria. However, four participants were lost to follow-up and did not complete the interventions. Since the study relied solely on in-person sessions and did not include a home program component, all remaining participants adhered to the treatment protocol (Figure 1). Table 2 shows the patients’ baseline demographic data for all groups. The mean age of the participants was approximately 47 years. The participants were categorized under obesity class 1 and experienced moderate pain intensity lasting for ≥10 months. There were no significant differences in any of the baseline parameters between groups.

### 3.2. HRV

After 6 weeks, the RR interval in the t-VNS group increased compared to that in the HRV-BF (mean difference [MD] = 53.0 ms; *p* = 0.037) group, indicating a longer time of rest between heartbeats. No differences were found between the SC-PT and t-VNS or HRV-BF groups. For the average HR, the t-VNS group demonstrated lower values compared to the HRV-BF (MD = −5.4 bpm; *p* = 0.039) group, indicating a more relaxed heart. No differences were found in this parameter between the SC-PT and t-VNS or HRV-BF groups. No other parameters of HRV (Rmssd, LF, HF, or LF/HF ratio) showed differences between any of the groups (Table 3).

### 3.3. VAS

After 6 weeks of intervention, the pain intensity in the t-VNS group decreased more than that in the HRV-BF (MD = −0.8 cm; *p* = 0.044) and SC-PT (MD = −0.9 cm; *p* = 0.018) groups. These differences exceeded the minimum clinically important difference (MCID) of 0.46 cm [34]. No significant differences were observed between the HRV-BF and SC-PT groups (MD = −0.1 cm; *p* = 0.694), indicating that the treatment effect did not exceed the MCID (Table 3).

### 3.4. PPT

Interestingly, the PPT in the most painful area of the neck was higher in the t-VNS group than in the groups that received HRV-BF (MD = 94.4 kPa; *p* < 0.001) or SCPT (MD = 56.2 kPa; *p* = 0.001). This difference exceeds the minimal detectable change (MDC) of 47.2 kPa [38]. The PPT increased in the SC-PT group compared to that in the HRV-BF group (MD = −38.2 kPa; *p* = 0.030), but this difference was smaller than the MDC (Table 3).

### 3.5. NDI

The t-VNS group demonstrated a more substantial reduction in disability than the HRV-BF (MD = −4.0; *p* = 0.015) and SC-PT (MD = −5.9; *p* < 0.001) groups, surpassing the established cutoff of 3.5 [39]. No significant differences were observed between the HRV-BF and SC-PT groups (MD = −1.9; *p* = 0.521), indicating that the treatment effect did not exceed the cutoff score (Table 3).

## 4. Discussion

This RCT evaluated the impact of ANS modulation on HRV, pain, and neck disability in participants with chronic neck pain. Participants receiving t-VNS for 6 weeks experienced significant improvements in certain HRV parameters, pain intensity, mechanical pain, and neck disability compared with those receiving HRV-BF or SC-PT. Owing to the limited availability of directly comparable research, we contextualized our findings within a broader framework of studies on other musculoskeletal conditions.

The current study demonstrates the effect of t-VNS on autonomic function through HRV, mainly affecting the RR interval and average HR. Our study shows that the RR interval in the t-VNS group increased significantly compared to that in the HRV-BF group, indicating a longer rest time between heartbeats. The observed increase in the RR interval, indicative of heightened parasympathetic (vagal) activity, supports the notion that t-VNS effectively modulates the ANS. These findings align with previous research demonstrating the ability of VNS to influence the balance between sympathetic and parasympathetic tone [40]. VNS triggers the parasympathetic nervous system, which affects the sinoatrial nodes and lowers HR [41].

Consistent with the observed increase in the RR interval, the t-VNS group exhibited a significant reduction in the average HR. This finding corroborates previous research indicating that VNS can decrease the HR by enhancing vagal efferent activity. This reduction in HR further supports the hypothesis that t-VNS effectively modulates ANS function, promoting a shift towards parasympathetic dominance [42].

In the current study, the t-VNS reduced pain intensity (as measured by the VAS) more effectively than both HRV-BF (MD = −0.8 cm) and SC-PT (MD = −0.9 cm). This effect was similar to that observed in previous studies on chronic low back pain [43], chronic knee osteoarthritis [44], fibromyalgia [45], and polymyalgia rheumatica [46]. Furthermore, t-VNS led to a reduction in mechanical pain, as demonstrated by the higher PPT values compared to both HRV-BF (MD = 94.4 kPa) and SC-PT (MD = 56.2 kPa). These findings are consistent with those of previous studies on chronic knee OA [44] and healthy volunteers [47]. Although the PPT increased in the SC-PT group compared to the HRV-BF group (mean difference = −38.2 kPa), this difference was below the MDC, suggesting a less pronounced effect. t-VNS can modulate pain perception by influencing both the central pain pathways and the ANS. This mechanism involves the activation of the vagus nerve, which triggers descending pain inhibitory pathways and regulates the release of neurotransmitters involved in pain modulation, such as norepinephrine and serotonin [48]. This neural pathway underscores the therapeutic potential of t-VNS in pain management by reducing pain sensitivity and increasing the PPT.

This study found that t-VNS substantially reduced neck disability, as demonstrated by the lower NDI score, compared to both HRV-BF (MD = −4.0) and SC-PT (MD = −5.9). These findings highlight the potential of t-VNS as a novel, non-invasive intervention for improving neck disability. The observed decrease in the NDI scores is consistent with previous research, emphasizing the therapeutic potential of t-VNS for modulating pain perception. The vagus nerve regulates pain and inflammatory pathways through its interaction with the ANS [49]. We likely modulated these pathways via VNS, which subsequently reduced neck-related disability.

Several mechanisms may contribute to the beneficial effects of t-VNS, as observed in this study. One theory posits that t-VNS mitigates sympathetic hyperactivity, a common feature of chronic pain, while concurrently augmenting parasympathetic activation. This dual modulation could potentially lead to a reduction in muscle tension and a more relaxed state [50]. Another proposed mechanism suggests that t-VNS modulates pain pathways by influencing the activity of key brain regions such as the locus coeruleus and nucleus raphe, which play pivotal roles in pain perception and modulation [51]. A reduction in inflammation is another proposed mechanism [52,53]. The t-VNS can activate the “inflammatory reflex”, a neural circuit that inhibits the release of pro-inflammatory cytokines that is often associated with chronic musculoskeletal pain [53].

HRV-BF enhances autonomic regulation by modulating the ANS to achieve a balance between sympathetic and parasympathetic activity. This is achieved by promoting cardiac coherence and enhancing baroreflex sensitivity. Ultimately, this can lead to reduced pain perception by mitigating stress and anxiety and improving neurovisceral integration, which facilitates communication between the heart and brain [54,55].

Despite these encouraging findings, it is essential to acknowledge the limitations of this study. Firstly, the short-term assessment of HRV may not fully capture the long-term effects of the interventions. Extending the assessment period to 12 and 24 h could provide a more comprehensive understanding of HRV modulation. Secondly, a longer follow-up period is necessary to evaluate the long-term efficacy of these interventions. Additionally, a lack of detailed participant information, including smoking habits, physical activity levels, sleep quality, and psychological history, could potentially influence HRV readings and should be considered when interpreting the results. Finally, the reliance on patient-reported outcomes (VAS and NDI) and the lack of blinding in the SC-PT sessions may have introduced potential bias into the study. To further enhance the understanding and application of t-VNS and HRV-BF, future research should focus on extending the measurement period of HRV to 12 h or 24 h; conducting extended follow-up studies to assess sustained benefits; collecting comprehensive information on lifestyle factors to identify potential confounding variables; incorporating objective measures, such as biomarker assessments and neurophysiological measures, to reduce bias; and implementing blind designs to minimize bias from both participants and therapists.

## 5. Conclusions

t-VNS appeared to be superior to HRV-BF and SC-PT in regulating certain HRV parameters, reducing neck pain, and improving functional capabilities. Consequently, it may serve as a novel and promising approach for the management of chronic neck pain, potentially providing a cost-effective, safe, accessible, user-friendly, and non-invasive alternative to conventional therapies. Further research is needed to explore the long-term effects and broader applications of t-VNS in the treatment of musculoskeletal pain. This research should involve careful patient selection, personalized treatment plans, regular follow-up monitoring, and comprehensive training for both patients and clinicians on the use of t-VNS devices.

## Figures and Tables

**Table 1:** Parameters of heart rate variability extracted from Polar device.

Parameter	Unit	Description
RR interval	ms	The interval between two consecutive heartbeats. A longer mean RR interval indicates a lower heart rate and is associated with increased parasympathetic activity.
HR	bpm	The number of times the heartbeats per minute. A lower resting heart rate often indicates more efficient heart function and improved cardiovascular fitness.
RMSSD	ms	This captures rapid beat-to-beat fluctuations in the RR interval, providing an estimate of vagally mediated changes in the HRV.
LF	ms^2^	This reflects baroreflex activity which buffer blood pressure changes.
HF	ms^2^	This reflects the short-term oscillation of the HR made by parasympathetic activity.
LF/HF ratio	number	This reflects the relative balance between sympathetic and vagal control.

Abbreviations: RR: interbeat interval; HR: heart rate; RMMSSD: root mean square of successive differences between normal heartbeats; LF: low frequency; HF: high frequency; LF/HF: ratio of LF to HF.

**Table 2:** Baseline characteristics of patients by group.

	t-VNS + SC-PT (n = 34)	HRV-BF + SC-PT (n = 34)	SC-PT Alone (n = 34)	*p*-Value
Gender (male/female) (n)	20/14	17/17	18/16	0.764
Age (years)	47.8 ± 11.1	47.1 ± 11.8	46.7 ± 10	0.921
Body mass index (kg/m^2^)	30.9 ± 5.4	31.9 ± 6.9	33.5 ± 7.2	0.248
Chronicity (weeks)	54.4 ± 27.3	49.9 ± 28.5	40.5 ± 28.6	0.121
Visual analog scale (cm)	6.1 ± 1.7	6.3 ± 1.7	6.4 ± 1.6	0.714
Resting heart rate (bpm)	78 ± 10	79 ± 10	81 ± 9	0.662
Resting systolic BP (mmHg)	126 ± 19	130 ± 15	126 ± 10	0.387
Resting diastolic BP (mmHg)	77 ± 9	80 ± 7	80 ± 7	0.207

Abbreviations: t-VNS: transcutaneous vagus nerve stimulation, SC-PT: standard-care physiotherapy, HRV-BF: heart rate variability biofeedback, SD: standard deviation, BP: blood pressure. All data are expressed as mean ± standard deviation, except gender, which is expressed as frequency.

**Table 3:** Baseline and comparison of heart rate variability parameters, pain, pressure pain threshold, and neck disability between groups.

	Baseline		Differences After 6 Weeks		
Variable	Groups	Mean ± SD	Groups	Mean Difference (95% CI)	*p*-Value
RR interval (ms)	t-VNS	789.9 ± 111.4	t-VNS vs. HRV-BF	53.0 (24.7, 70.9)	0.037 *
	HRV-BF	781.6 ± 110.1	t-VNS vs. SC-PT	30.6 (−20.7, 81.9)	0.447
	SC-PT	765.1 ± 92.5	HRV-BF vs. SC-PT	−22.4 (−73.4, 28.6)	0.861
Average HR (bpm)	t-VNS	78.4 ± 10.3	t-VNS vs. HRV-BF	−5.4 (−10.6, −0.2)	0.039 *
	HRV-BF	79.2 ± 10.0	t-VNS vs. SC-PT	−3.4 (−8.7, 1.9)	0.366
	SC-PT	80.6 ± 8.9	HRV-BF vs. SC-PT	2.0 (−3.2, 7.3)	1.000
Rmssd (ms)	t-VNS	37.1 ± 14.6	t-VNS vs. HRV-BF	0.1 (−7.6, 7.8)	1.000
	HRV-BF	35.7 ± 14.0	t-VNS vs. SC-PT	3.0 (−4.8, 10.8)	1.000
	SC-PT	40.9 ± 14.4	HRV-BF vs. SC-PT	2.9 (−5.0, 10.7)	1.000
LF (ms^2^)	t-VNS	428.1 ± 360.3	t-VNS vs. HRV-BF	1.0 (−14.1, 16.0)	0.987
	HRV-BF	299.2 ± 209.3	t-VNS vs. SC-PT	8.3 (−6.7, 23.3)	0.392
	SC-PT	525.8 ± 383.7	HRV-BF vs. SC-PT	7.3 (−7.7, 22.4)	0.481
HF (ms^2^)	t-VNS	411.1 ± 379.2	t-VNS vs. HRV-BF	−3.4 (−19.6, 12.8)	0.872
	HRV-BF	248.6 ± 307.4	t-VNS vs. SC-PT	1.1 (−15.2, 17.3)	0.987
	SC-PT	500.2 ± 328.5	HRV-BF vs. SC-PT	4.5 (−11.8, 20.7)	0.790
LF/HF ratio	t-VNS	1.2 ± 0.7	t-VNS vs. HRV-BF	0.3 (−0.1, 0.8)	0.276
	HRV-BF	1.0 ± 0.5	t-VNS vs. SC-PT	0.4 (−0.2, 0.7)	0.308
	SC-PT	1.2 ± 0.5	HRV-BF vs. SC-PT	−0.1 (−0.5, 0.4)	1.000
VAS (cm)	t-VNS	6.1 ± 1.1	t-VNS vs. HRV-BF	−0.8 (−1.5, −0.2)	0.044 *
	HRV-BF	6.2 ± 1.7	t-VNS vs. SC-PT	−0.9 (−1.7, −0.2)	0.018 *
	SC-PT	6.4 ± 1.6	HRV-BF vs. SC-PT	−0.1 (−0.9, 0.6)	0.694
PPT (kPa)	t-VNS	227.5 ± 70.0	t-VNS vs. HRV-BF	94.4 (58.6, 130.3)	<0.001 *
	HRV-BF	285.6 ± 117.0	t-VNS vs. SC-PT	56.2 (20.6, 91.8)	0.001 *
	SC-PT	256.5 ± 113.6	HRV-BF vs. SC-PT	−38.2 (−73.8, −20.7)	0.030 *
NDI (/50)	t-VNS	21.0 ± 6.0	t-VNS vs. HRV-BF	−4.0 (−7.3, −0.6)	0.015 *
	HRV-BF	21.5 ± 6.1	t-VNS vs. SC-PT	−5.9 (−9.3, −2.5)	<0.001 *
	SC-PT	22.1 ± 4.7	HRV-BF vs. SC-PT	−1.9 (−5.3, 1.5)	0.521

Abbreviations: SD: standard deviation, RR interval: time elapsed between two successive R-waves of QRS signal on electrocardiogram, t-VNS: transcutaneous vagus nerve stimulation, HRV-BF: heart rate variability biofeedback, SC-PT: standard care physiotherapy, HR: heart rate, Rmssd: root mean square of successive differences between normal heartbeats, LF: absolute power of low-frequency band, HF: absolute power of high-frequency band, VAS: visual analog scale, PPT: pressure pain threshold, NDI: neck disability index, 95% CI: 95% confidence interval. * Statistically significant.

## Data Availability

The original contributions presented in this study are included in the article; further inquiries can be directed to the corresponding author.
